# Insights for conducting real-time focus groups online using a web conferencing service

**DOI:** 10.12688/f1000research.10427.1

**Published:** 2017-02-09

**Authors:** James Kite, Philayrath Phongsavan

**Affiliations:** 1 Prevention Research Collaboration, Sydney School of Public Health and Charles Perkins Centre, University of Sydney, NSW, Australia

**Keywords:** online research, focus groups, web conferencing, methodology, critical reflection

## Abstract

**Background**

Online focus groups have been increasing in use over the last 2 decades, including in biomedical and health-related research. However, most of this research has made use of text-based services such as email, discussion boards, and chat rooms, which do not replicate the experience of face-to-face focus groups. Web conferencing services have the potential to more closely match the face-to-face focus group experience, including important visual and aural cues. This paper provides critical reflections on using a web conferencing service to conduct online focus groups.

**Methods**

As part of a broader study, we conducted both online and face-to-face focus groups with participants. The online groups were conducted in real-time using the web conferencing service, Blackboard Collaborate ^TM^. We used reflective practice to assess how the conduct and content of the groups were similar and how they differed across the two platforms.

**Results**

We found that further research using such services is warranted, particularly when working with hard-to-reach or geographically dispersed populations. The level of discussion and the quality of the data obtained was similar to that found in face-to-face groups. However, some issues remain, particularly in relation to managing technical issues experienced by participants and ensuring adequate recording quality to facilitate transcription and analysis.

**Conclusions**

Our experience with using web conferencing for online focus groups suggests that they have the potential to offer a realistic and comparable alternative to face-to-face focus groups, especially for geographically dispersed populations such as rural and remote health practitioners. Further testing of these services is warranted but researchers should carefully consider the service they use to minimise the impact of technical difficulties.

## Introduction

Focus groups are a well-established qualitative research methodology that have become increasingly popular among social researchers over the last few decades ^1, 2^. Their popularity is tied to their ability to use group interactions to elicit detailed responses, which have been shaped as much by social cues as by the individual’s own beliefs and perceptions ^3– 5^. However, traditional face-to-face focus groups have some disadvantages, particularly when dealing with hard-to-reach or geographically dispersed populations and sensitive topics ^6– 10^. Increasingly, the Internet offers a real alternative to face-to-face groups as technology improves and connection spreads. Online focus groups therefore have the potential to address this gap while also offering researchers the opportunity to avoid the costs of finding an ideal location to host their groups ^7, 11^.

Using online platforms for focus groups has been trialled over the last 20 years with an increasing number of studies making use of asynchronous platforms (e.g. email and discussion boards) for their research ^8, 9, 11, 12^. This includes research with rural and remote nurses ^4^, travelling nurses ^13^, and gay and bisexual men with cancer ^10^. There are a number of potential benefits for conducting research in this way, including increased speed of data collection, lower cost, and, of particular relevance to biomedical and health-related research, improved opportunity for some population groups to participate in research ^2, 14– 16^. However, using text-based platforms changes the nature of focus groups, with the major criticisms being that you lose spontaneity in participant responses and also visual and aural cues, which collectively promote the expression of emotions and can be very influential in directing participant interactions ^2, 3, 8^. Some researchers have made use of chat services to run synchronous (i.e. real-time) online focus groups [for example ^8^] but again the visual and aural cues are lost, and participants and moderators must be skilled in reading and writing to be able to respond quickly while also being as unambiguous as possible in order to avoid misunderstandings ^11^. Chat-based focus groups also come with a risk of returning inadequate data quality as participants and moderators take short-cuts to speed up writing ^2, 17^.

Audio-visual tools, such as web conferencing services, offer a way of more closely mirroring the experience of a face-to-face focus group but this appears to be an under-used approach. Indeed, we found only two studies, both published in 2015, that report on the experience of conducting focus groups in this way ^11, 13^. This is likely because, until recently, limited bandwidth and inappropriate or inadequate platforms meant that online face-to-face groups faced significant technical barriers. This is no longer the case with Internet penetration and speed accelerating rapidly, especially in developing nations ^18^. In addition, and importantly, there is research that suggests that social interactions are similar in both the face-to-face and online environments ^19^, indicating that online face-to-face focus groups may be a viable alternative to traditional groups. To date, however, there is little available evidence on the experience of conducting groups in this way. To address this gap, we report on our experience of conducting focus groups using a web conferencing service, with comparisons to traditional face-to-face focus groups where relevant.

## Methods

We recently conducted an evaluation of a postgraduate subject in public health at the University of Sydney, approved by the University of Sydney Human Research Ethics Committee (Project No. 2014/1015). In the unit being evaluated, students have the choice of studying either face-to-face or online, with most students who complete the unit online either living a significant distance from campus (e.g. interstate) or having other commitments (e.g. full-time work) that make it difficult to attend face-to-face classes. Although the unit content is the same regardless of the delivery mode, we hypothesised that the student learning experience would be significantly different, making it important that both face-to-face students and online students had an equal opportunity to participate in this study. One part of the evaluation study involved focus groups with former and current students and so, to ensure equal opportunity to participate, we offered both face-to-face and online focus groups. The online focus groups were completed in real-time over a web conferencing service, Blackboard Collaborate ^TM^ (Version 9.1; http://www.blackboard.com/online-collaborative-learning/).

Participants were recruited via email in a two-stage process. The first stage involved seeking expressions of interest in participating in the research from all students who completed the subject in either 2013 or 2014 (n=400). Emails were sent to their official University email accounts. The second stage required those who had expressed interest (n=23) to complete a short survey (see Supplementary material) on their preferred focus group platform, as well as time and date availability. Two participants had to withdraw after this second stage as it was not possible to accommodate their schedules.

In total, we conducted 5 focus groups: 3 face-to-face (two groups with n=4 and one with n=6 participants) and 2 online (n=3 and n=4 participants respectively). The focus groups ran for approximately 90 minutes but online participants were encouraged to logon before the scheduled start of the focus group to allow time to calibrate microphones and cameras if required. Additionally, in the invitation to the focus group, online participants had been directed to a Help page ( http://sydney.edu.au/elearning/staff/help/collaborateHelp.shtml) where they could trial Blackboard Collaborate ^TM^ and ensure that their system met the minimum requirements for running this service.

Moderation of all groups was conducted by the same person (JK), with an effort made to keep the style of moderation similar in both online and face-to-face groups. This included allowing multiple speakers at the same time during online groups, as opposed to setting Collaborate ^TM^ to only allow one speaker at a time. The topics for discussion, which were the same in both the online and face-to-face focus groups, focused on assessment practices within the subject but also canvassed experiences with tutorials and lectures (see Supplementary material for complete discussion guide). The only difference between the online and face-to-face focus groups was that some time was allocated at the beginning of the online focus groups to provide a brief tutorial on using some of Collaborate’s ^TM^ features. This paper does not report the findings from these focus groups; these are available elsewhere ^20^.

We used reflective practice to assess how the conduct and content of the groups were similar and how they differed across the two platforms ^21^. This involved reviewing the audio recordings and transcripts from the focus groups and assessing what happened, reflecting on our conduct and interaction with the groups, and what we would do differently next time. In particular, we reflected on our own experience (i.e. as researchers) with managing and moderating the groups. All of the reflections presented here are based on our impression of how the groups functioned and what we perceived as being the most salient issues that arose throughout. Although we did not directly ask the participants about their experience in either format of focus group, we were also able to glean some insights from remarks they made during the conduct of the focus group.

## Participants’ and researchers’ experience

When we were arranging the groups, potential participants were asked to nominate their preferred platform for the focus group. Based on responses to the survey, there was considerable interest (n=17 of 23 responses) in conducting online groups, especially among those who had work or family commitments that made attending face-to-face focus groups more difficult. Indeed, some of the online participants made mention of the fact that they were grateful of being given the opportunity to put forward their views in such a forum, something that would not have been possible had we only conducted face-to-face groups.

*“Thanks for doing this. Interesting to know subjects seriously evaluating how they do things.”*

The advantage of using a web conferencing service compared to a chat service to run synchronous focus groups online is that it more closely mirrors the experience of face-to-face groups: participants are able to respond to visual and aural clues that would otherwise be missed. This was certainly evident in the online focus groups; our perception was that the interaction between participants and the moderator was dynamic and similar to that experienced in the face-to-face groups. Participants were genuinely engaged and attentive, although personal issues (e.g. distractions from phones, children, and other background noise) did occasionally interrupt discussions. Further, we noted that communication was considerably slower and more time was spent discussing issues of no relevance to the research, compared to the face-to-face groups. In particular, online participants spent some time familiarising themselves and each other with the web conferencing service, as well as discussing the novelty of web conferencing service and any technical difficulties they had experienced or were experiencing, as shown in the following example.

*      Participant 1: “[I think the] mic is bad. This is not working well.”*
*      Participant 2: “We can hear you alright but it is just cutting out.”*
*                           Participant 1: “I’ll try logging off.”*

Although the slower and more distracted discussion did produce less data overall ^20^, the quality of the data we did obtain was of an equal to that in the face-to-face groups, which is in line with the experience of Abrams *et al.* ^11^. In general, the themes that emerged from both the face-to-face and online groups were similar but it was obvious that there were critical differences in the student experience between online and face-to-face students. By way of example, a group assignment was discussed at length in all of the focus groups but the difference in experience for face-to-face and online students could not have been starker. The face-to-face students praised it but the online students had a deeply negative experience. This contrast may have been missed had we not conducted the online focus groups.

Importantly, the online groups did function as a focus group should; that is, there was genuine discussion *between* participants, rather than just between a participant and an interviewer, as you would find in a group interview ^5^. The groups still provided important and valuable insights even though they did not cover as many topics as the face-to-face groups. In recognition of this, in the second group the moderator focused more on topics where we expected the experience of online and face-to-face students to be most divergent (e.g. tutorials and group work) and less on experiences that were likely to be similar (e.g. written assignments). This change did not involve any modification to the discussion guide, only a change in the time allocated to each section.

The experience of moderation was relatively similar across both platforms but there were some minor differences. In particular, the moderator had to be familiar with Collaborate ^TM^ in order to be able to quickly troubleshoot with participants when necessary, something that was obviously not necessary for the face-to-face groups. This included having to deal with participants who were using the chat feature when their microphones were not working. Having one participant contributing to the discussions via chat did add a layer of complexity and meant that the moderator had to allow additional time for the participant to type and for all participants to read and react to the response. However, this did not appear to affect the conversation in any meaningful way, although it is possible that participants using chat features may have been taking short cuts to speed up writing, as noted in previous research ^2, 17^. It is also worth noting that it was not necessary for the moderator to alter style or speed of talking as participants could generally hear the conversation clearly, as they would in a face-to-face group.

We noted that online participants were more inclined to withdraw from the study. We had originally recruited 5 participants for each focus group but 3 withdrew (2 before group 1 and 1 before group 2) in the hour before their scheduled focus group was due to commence. Further, 2 more participants withdrew (1 in each group) after the groups commenced, with one having technical difficulties and the other because of constant distractions from their children. In contrast, only 1 participant withdrew from the face-to-face groups.

Finally, sound quality was a significant issue, specifically for transcription purposes. Participants did experience some difficulty hearing each other during the groups but this was not a major problem. However, when it came time to transcribe the recordings, echoing made it extremely difficult to do so accurately.

## Discussion

We found that the use of a web conferencing service to conduct focus groups has potential, even though there are a number of issues. It is worth highlighting, however, the thankfulness reported by participants in the online groups, and the fact that there were some critical differences in experience between online and face-to-face students that may have been missed without conducting the online groups. Although some may argue that we could have captured the views of online students through other means like a survey, such an approach would have meant losing the social interactions that are a key feature of focus groups. This highlights the importance of continuing to trial new technologies so that hard-to-reach groups are given greater opportunity to participate in all types of research.

It was not unexpected that participants spent considerable online focus group time discussing technical difficulties they were experience or familiarising with the technologies. To circumvent this, we directed participants to the Help page in the participation information and encouraged participants to logon early, both of which were done by at least some participants, although it was not clear whether all participants made use of these options. We also allocated time at the beginning of the focus group to providing a brief tutorial on Collaborate ^TM^ but, despite all this, significant time was still spent discussing the service itself at the expense of the research topics. This suggests that more may need to be done to address this issue, which may include, for example, scheduling more time for online focus groups than for comparable face-to-face groups. Alternatively, other researchers planning on using a web conferencing service could consider scheduling fewer topics for discussion.

We had selected Blackboard Collaborate ^TM^ as the web conferencing service because it is supported by the University’s online learning management system, with which participants would be familiar. Using this service also meant that participants could access it without needing to create additional online profiles and did not need to download any software. Collaborate ^TM^ also offers the ability to upload slides, which can be viewed and edited by participants during the session, a feature not offered by some other web conferencing services. It also includes an in-built recording system, which means that researchers can avoid the need to source or purchase a stand-alone recording device and the need to ensure sufficient battery life in order to record the entire discussion. The service also provided prompts to begin recording, reducing the risk of missing any of the discussion by mistake. However, a downside was that, although participants were familiar with the online learning management system, they were unfamiliar with Collaborate ^TM^ as the University had only recently begun supporting it. One participant who had had considerable trouble joining the focus group even asked why we had not used a more familiar service like Skype. Researchers considering using online focus groups should consider following the lead of Tuttas ^13^ and evaluate several available web conferencing services when designing their study.

Similar to our experience, Tuttas ^13^ experienced issues with sound quality but resolved it by asking participants to mute their microphones when they were not speaking. This may be advisable as standard practice for other researchers, at least until the technology improves. That said, this would encourage participants to take turns to speak and therefore might reduce the dynamic nature of the discussion. This risks making the group less like a face-to-face focus group and more like a group interview ^5, 11^. An alternative may be to ask all participants to use headsets with a microphone, rather than relying on their computer’s in-built speakers and microphone. The use of headsets would eliminate echoing, improving the quality of recording.

The phenomenon of online participants being more likely to withdraw was also noted by Tuttas ^13^, suggesting that there is something about the online environment that reduces the connection between participants and research. One of the benefits of online research is that participants can feel an increased sense of anonymity and may therefore be more willing to offer their opinion ^14, 15^. It may be, however, that this feeling of anonymity reduces participants’ connection with the research and makes them more likely to disengage. Tuttas ^13^ recommends that researchers over-sample in order to compensate for this attrition, which we echo but with the caveat that the smaller group sizes that we had were easier to manage in the online environment. Had these participants not withdrawn, we believe the larger group sizes would have further reduced the number of topics covered. Researchers may therefore find it worthwhile to plan for more online focus groups with fewer participants than they would if conducting face-to-face groups.

Online focus groups are being used within biomedical and health-related research, usually to enable increased participant anonymity when discussing sensitive topics or to bring together hard-to-reach populations ^4, 10, 13^. Their potential is also recognised in advertising research ^16^, which has implications for social researchers interested in the impact of exposure to advertising on health. However, these studies have recognised the limitations of text-based platforms, which include difficulty in organising and managing real-time groups, the need for motivated participants in asynchronous groups in order to maintain participation over several days or weeks, and the exclusion of participants with low literacy levels. Our experience suggests that web conferencing services offer a viable alternative to face-to-face focus groups and are worthy of further testing. Importantly, they will overcome the barriers inherent in text-based groups, which will strengthen research methodology.

The major limitation of this paper is that we had not put any formal reflective process in place before conducting the focus groups because we had not intended to explore the use of online focus groups before we conducted them. This has meant that we were only able to take into account our own perceptions and experience with the groups and not that of the participants. Nonetheless, we feel that these reflections are potentially valuable for researchers interested in using this methodology because, to date, very little is available to guide the implementation of online focus groups. More formal testing of this method is needed but our reflections should help to improve their design and implementation while this testing is being carried out.

## Conclusions

Our experience with using a web conferencing service to conduct a real-time focus group was mixed. Online services have the potential to offer a realistic and comparable alternative to face-to-face focus groups for geographically dispersed populations. Further testing of available services is certainly warranted. However, technical difficulties, particularly with ease of participant access and poor recording quality, mean that we strongly recommend that researchers carefully consider and test the web conferencing service that they intend to use for hosting their focus groups. 

## Data availability

The data referenced by this article are under copyright with the following copyright statement: Copyright: © 2017 Kite J and Phongsavan P

The qualitative data underpinning this analysis is not available because it cannot be sufficiently anonymised.
