# Supplementary material for: Insights for conducting real-time focus groups online using a web conferencing service
Source: F1000Res. 2017 Feb 9;6:122. [Version 1] doi: 10.12688/f1000research.10427.1 (PMC5527981; doi:10.12688/f1000research.10427.1)
Supplement: Supplementary file 1 [file f1000research-6-11236-s0000.tgz › 5b0a95ec-9fe7-4ba0-b83c-8178202606c7.docx]

# Focus group survey

Thank you for your interest in participating in our research, which aims to improve assessment practices in [the subject]. This survey seeks your preferences on focus group delivery mode and time. Please note that completing the survey does not lock you into participating in the research; you can change your mind later. If you have any questions about any aspect of the research, you can contact James Kite ([email address]) at any time.

You have a choice of participating in the focus group in person at the University of Sydney's Camperdown campus or online. In either case, the focus group will last for approximately 90 minutes. Please note that the exact venues and times will be dependent on the preferences of all participants. Every effort will be made to accommodate you but there is a possibility that your preferred delivery mode (in person or online) or time will not go ahead if there is not enough participants to fill that group.

1. Would you prefer to complete the focus group in person or online?
   1. In person
   2. Online is preferred but I could attend in person if the time is suitable
   3. I can only participate online
   4. I am not interested in participating
2. Will you be available to participate in a focus group during the following periods? You should tick 'Yes' even if you will only be available for part of the period.
   1. Monday, 19 January - Friday, 23 January 2015
   2. Tuesday, 27 January - Sunday, 1 February 2015
   3. Monday, 2 February - Sunday, 8 February 2015
3. What days and times would you prefer to participate during the period Monday, 19 January - Friday, 23 January? Tick all that apply.

|  | Morning (9am-12pm) | Afternoon (12pm-5pm) | Early evening (5pm-8pm) |
| --- | --- | --- | --- |
| Monday, 19 Jan |  |  |  |
| Tuesday, 20 Jan |  |  |  |
| Wednesday, 21 Jan |  |  |  |
| Thursday, 22 Jan |  |  |  |
| Friday, 23 Jan |  |  |  |

1. What days and times would you prefer to participate during the period Tuesday, 27 January - Sunday, 1 February? Tick all that apply.

|  | Morning (9am-12pm) | Afternoon (12pm-5pm) | Early evening (5pm-8pm) |
| --- | --- | --- | --- |
| Tuesday, 27 Jan |  |  |  |
| Wednesday, 28 Jan |  |  |  |
| Thursday, 29 Jan |  |  |  |
| Friday, 30 Jan |  |  |  |
| Saturday, 31 Jan |  |  |  |
| Sunday, 1 Feb |  |  |  |

1. What days and times would you prefer to participate during the period Monday, 2 February - Sunday, 8 February? Tick all that apply.

|  | Morning (9am-12pm) | Afternoon (12pm-5pm) | Early evening (5pm-8pm) |
| --- | --- | --- | --- |
| Monday, 2 Feb |  |  |  |
| Tuesday, 3 Feb |  |  |  |
| Wednesday, 4 Feb |  |  |  |
| Thursday, 5 Feb |  |  |  |
| Friday, 6 Feb |  |  |  |
| Saturday, 7 Feb |  |  |  |
| Sunday, 8 Feb |  |  |  |

1. Name:
2. Do you have any comments you would like to make regarding your availability for the focus groups?

Thank you for completing the survey. You will hear from James to confirm your focus group time and venue shortly.

# Focus group discussion guide

## Introduction/housekeeping (5-10 mins)

*Welcome to everyone – hand out vouchers, consent forms for signatures.*

*Purpose of research is to inform assessment practices within [the subject]. This phase of the research is being conducted with former students of [the subject]; later phases will look at perceptions and performance of current students under the revised assessment process. A summary of results can be provided to you if you’re interested.*

*Housekeeping:*

*(online) Quick guide to using Collaborate:*

## Icebreaker (5 mins)

*Ask everyone for their names, what they were or are studying and why, how they studied the subject, and 1 thing they remember about it*

*Remind everyone about the subject’s structure, including assessments*

## Experience with assessment in higher education (30 mins)

- What things stand out to you with regards to assessment in the subject?
  - Relevance of assessment to course, degree, and future – Were there clear links between what you did for assessment and what was addressed in the workshops and tutorials?
  - Design/structure of the assessments
  - Understanding of expectations
  - Assessment feedback
- How does assessment in the subject compare with your experience in higher education more broadly?
  - Good/bad experiences with assessment
  - What kinds of assessments do you like/dislike or find most challenging/rewarding?
  - Anything that you think would be suitable for the subject?

## Assignment 1 and rubric (25 mins)

- Thoughts on the new rubric
  - Good things/bad things about it
  - Change how you would have approached the assignment?
  - Would it have made dealing with grades and marker feedback better/worse/no different?
  - Is it clear how the criteria and standards relate to one another and to the course aims and learning objectives?
  - Will this help future students?
- Thoughts on the exemplars/marking exercise
  - Helpful in understanding your own work?
  - How did you use the rubric in marking?
  - Would a tutor explanation of the rubric and how assignments will be marked make things better/worse/no different?
  - Would this approach be helpful for future students?
  - Would you like to do this exercise for all assessments or would 1 be enough for you to apply the lessons to other assessments?

## New assignment (15 mins)

- What do you remember about the group-based tutorial work?
  - Likes/dislikes
  - Group work vs individual work
  - Peer assessment process

*Provide brief explanation of new assignment*

- Thoughts on new assignment?
  - What should the ‘something’ be? Presentation, report, project plan?
  - Should it be group work? If it is group work, how should the marks be allocated i.e. 1 mark for everyone in the group or at least some part individually marked? Should there be peer assessment?
  - Suitability for online and face-to-face? Would different styles of assessment be ok for the different delivery modes?

## Wrap up (5-10 mins)

- Ideas for changing assessment design, communication of expectations, and feedback in PUBH5033

*Thank participants and advise them of what the next steps for the research will be*
