# Supplementary material for: Insights for conducting real-time focus groups online using a web conferencing service
Source: F1000Res. 2017 Feb 9;6:122. [Version 1] doi: 10.12688/f1000research.10427.1 (PMC5527981; doi:10.12688/f1000research.10427.1)
Supplement: Peer review history

# Peer review: Referee response for version 1

**DOI:** 10.5256/f1000research.11236.r22904

**Published:** 2017-05-18

**Authors:** Marita Hefler

**Affiliations:** 1 Menzies School of Health Research, Darwin, NT, Australia

This is a useful article for researchers who use focus groups, particularly those for whom online approaches may assist with overcoming barriers of geography, mobility or time constraints to ensure inclusion of hard-to-reach groups.  Overall, it provides a number of useful tips and helpful discussion, however there are some issues which require clarification.

  

- It is unclear if the platform offered for focus group participation matched the mode of study that participants had completed. The second paragraph under the heading methods states that all students who completed the subject were contacted about participating in the research. Those who were interested were then asked about their preferred platform. There does not appear to have been any distinction made about whether students completed the study unit online or face to face. The first paragraph under the sub-heading ‘participants’ and researchers’ experience’ also seems to confirm this. However, the last part of the third paragraph under the same heading seems to suggest that participants were grouped for focus groups according to their mode of study – ie students who completed the unit face-to-face were assigned to face-to-face focus groups and online students to online focus groups. (“By way of example, a group assignment was discussed at length in all focus groups, but the difference in experience for face-to-face and online students could not have been starker…This contrast may have been missed had we not conducted the online focus groups”). Can the authors please clarify? If the mode of study was not matched to the focus group platform offered, the statement that the contrast would have been missed is not valid – it would be more a reflection of doing a sufficient number of focus groups, rather than offering participation through different platforms.
- Overall, I think the authors somewhat downplay the impact of several of the issues on the quality of data collected through online focus groups. The third paragraph on the second page suggests that use of web conferencing came close to approximating the experience of face-to-face groups, however the paragraph goes on to note that communication was slower, time was necessarily to resolve technical issues even after encouraging participants to log on and trouble shoot prior to commencement. Not only did this produce less data, but the issue of sound quality mentioned in the last paragraph on page two seems to also be significant – both because of the difficulty participants had hearing each other, and the difficulty of accurate transcription. In addition, one participant being forced to participate via chat would also have impacted on the aural and visual cues which the authors rightly note are an important component of focus groups.

There is discussion about online participants being more likely to withdraw (either before or during focus groups), however given the very small sample, this should be treated with caution. I would suggest the most salient findings in terms of withdrawal are the challenge of technical difficulties forcing withdrawal, and also the possibility of participants having outside distractions from wherever they are participating, which obviously don’t exist in a dedicated face-to-face focus group environment.

I have read this submission. I believe that I have an appropriate level of expertise to confirm that it is of an acceptable scientific standard, however I have significant reservations, as outlined above.

# Response

**Published:** 2017-05-28

**Authors:** James Kite

**Affiliations:** University of Sydney, Australia

Thank you very much for your review, Marita. We have outlined our responses to each of your specific queries below and revised the paper accordingly.

**Reviewer comment: **It is unclear if the platform offered for focus group participation matched the mode of study that participants had completed. The second paragraph under the heading methods states that all students who completed the subject were contacted about participating in the research. Those who were interested were then asked about their preferred platform. There does not appear to have been any distinction made about whether students completed the study unit online or face to face. The first paragraph under the sub-heading ‘participants’ and researchers’ experience’ also seems to confirm this. However, the last part of the third paragraph under the same heading seems to suggest that participants were grouped for focus groups according to their mode of study – ie students who completed the unit face-to-face were assigned to face-to-face focus groups and online students to online focus groups. (“By way of example, a group assignment was discussed at length in all focus groups, but the difference in experience for face-to-face and online students could not have been starker…This contrast may have been missed had we not conducted the online focus groups”). Can the authors please clarify? If the mode of study was not matched to the focus group platform offered, the statement that the contrast would have been missed is not valid – it would be more a reflection of doing a sufficient number of focus groups, rather than offering participation through different platforms.

*Authors’ response: Participants were not forced to complete the focus groups on the platform that matched their mode of study. However, only 2 of the participants who attended the face-to-face groups completed the subject online, while all of the participants who attended the online groups completed the subject online. As participant group platform did almost perfectly match the subject delivery mode, we believe that contrast is valid. We have modified the paper to make this detail clear.*

**Reviewer comment: **Overall, I think the authors somewhat downplay the impact of several of the issues on the quality of data collected through online focus groups. The third paragraph on the second page suggests that use of web conferencing came close to approximating the experience of face-to-face groups, however the paragraph goes on to note that communication was slower, time was necessarily to resolve technical issues even after encouraging participants to log on and trouble shoot prior to commencement. Not only did this produce less data, but the issue of sound quality mentioned in the last paragraph on page two seems to also be significant – both because of the difficulty participants had hearing each other, and the difficulty of accurate transcription. In addition, one participant being forced to participate via chat would also have impacted on the aural and visual cues which the authors rightly note are an important component of focus groups.

*Authors’ response: It was not our intention to downplay the significance of these issues and we have therefore tried to amend the language around the above points to make this clear. However, we have also clarified that the issue of participants being unable to hear each other during the groups was indeed minor and did not affect the flow of discussion.*

**Reviewer comment: **There is discussion about online participants being more likely to withdraw (either before or during focus groups), however given the very small sample, this should be treated with caution. I would suggest the most salient findings in terms of withdrawal are the challenge of technical difficulties forcing withdrawal, and also the possibility of participants having outside distractions from wherever they are participating, which obviously don’t exist in a dedicated face-to-face focus group environment.

*Authors’ response: We agree that the issues of technical difficulties and outside distractions that forced withdrawal are noteworthy because they would not affect face-to-face groups. We have added this point to the ‘Discussion’. We have also added a note regarding the sample size to the ‘Participants’ and researchers’ experience’ section.*

# Peer review: Referee response for version 1

**DOI:** 10.5256/f1000research.11236.r21467

**Published:** 2017-04-04

**Authors:** Sarah Collard, Edwin van Teijlingen

**Affiliations:** 1 Faculty of Health and Social Sciences, Bournemouth University, Bournemouth, UK; 2 Centre for Midwifery, Maternal & Perinatal Health, Faculty of Health & Social Sciences, , Bournemouth University, Bournemouth, UK

**Title and Abstract**: These are appropriate. There are grammatical errors within the abstract that need to be amended.

**Article content**: This article provided an interesting comparison of face-to-face and online focus groups. Using online conferencing is an important topic to explore and these findings can aid researchers in the positives about using online conferencing for hard to reach populations. It also will aid researchers as it provides concerns that arise in using such technology.

 It is always good to see researchers writing about their own experiences of applying (new) research methods, this gives students and junior researcher a chance to consider any issues with these methods and helps them in their decision to select appropriate ones for their own research.

 We have summarised the two major variants of internet-based focus groups:  (a) written chat; and (b) audio/video conferencing.  Our paper highlighted the differences and similarities of these two approaches, as well as the strengths and limitations of each internet-based FG methods ^1^.  We feel the authors could have made a little more effort to put their particular approach to conducting online focus groups in the wider field of online focus group techniques. 

 This article does need amendments as there are quite a lot of grammatical errors and inconsistencies, particularly in product names (e.g. Blackboard Collaborate and then it just says Collaborate, and then goes back to Blackboard Collaborate). In regards to the methods, this also needs more details. Reflective practice was used, but this needs more explanation of why this was chosen and the pros versus cons of this method. Was this the method to use?

 Questions also arise in the explanation of how the focus groups were conducted. For example, more explanation on privacy concerns needs to be discussed. Did you lay ground rules down with the participants? Why was privacy not taken into account prior to conducting the focus groups?

 Also, to help other researchers using online conferencing for focus groups, what are key concerns and positive techniques that should be used for future groups? In terms of results, less data were obtained from the online focus groups, why does this not mean there is a significant difference? Please justify why this is still a sufficient method. Regarding the technical issues, how might these be lessened for others?

 The major concerns throughout are the aspects that the analysis of this project was not thought of prior to conducting the research. If it was, this needs to be stated more clearly. In addition, please be careful of word choices (e.g. “thankfulness”) and grammar errors throughout. This will decrease confusion and allow it to read smoother.

**Conclusion**s: A bit more explanation about concerns and outcomes needs to be taken into consideration. Particularly after the statement that less data was obtained through the online version compared to face to face. This seems like a large difference, so please state how the online version is still beneficial besides the decrease in data obtained and technical difficulties that can create concern. The topic of this paper is of interest and can bring new insight, but it needs to be a bit more explicit in how.

We have read this submission. We believe that we have an appropriate level of expertise to confirm that it is of an acceptable scientific standard, however we have significant reservations, as outlined above.

## References

[1] CollardSvan TeijlingenE: Online focus group: New approaches to an ‘old’ research method. *Health Prospect*.2016;15(3) :4-7

# Response

**Published:** 2017-05-28

**Authors:** James Kite

**Affiliations:** University of Sydney, Australia

Thank you very much for your review. As well as uploading a revised version of our paper, we have outlined our responses to each of your specific queries below.

**Reviewer comment: **Title and Abstract: These are appropriate. There are grammatical errors within the abstract that need to be amended.

*Authors’ response: We have amended the abstract to try and address any grammatical errors.*

**Reviewer comment: **Article content: This article provided an interesting comparison of face-to-face and online focus groups. Using online conferencing is an important topic to explore and these findings can aid researchers in the positives about using online conferencing for hard to reach populations. It also will aid researchers as it provides concerns that arise in using such technology.

 It is always good to see researchers writing about their own experiences of applying (new) research methods, this gives students and junior researcher a chance to consider any issues with these methods and helps them in their decision to select appropriate ones for their own research.

*Authors’ response: Thank you. The principal motivation for writing this paper was to share our experience as we would have found this information to be of considerable benefit had it been available at the time we were designing the study. We hope it can be of use to others, as you have suggested.*

**Reviewer comment: **We have summarised the two major variants of internet-based focus groups:  (a) written chat; and (b) audio/video conferencing.  Our paper highlighted the differences and similarities of these two approaches, as well as the strengths and limitations of each internet-based FG methods.  We feel the authors could have made a little more effort to put their particular approach to conducting online focus groups in the wider field of online focus group techniques.

*Authors’ response: Thank you for sharing your paper. We have incorporated its findings into our introduction and discussion.*

**Reviewer comment: **This article does need amendments as there are quite a lot of grammatical errors and inconsistencies, particularly in product names (e.g. Blackboard Collaborate and then it just says Collaborate, and then goes back to Blackboard Collaborate).

*Authors’ response: We have tried to address any grammatical errors in the revised version.*

**Reviewer comment: **In regards to the methods, this also needs more details. Reflective practice was used, but this needs more explanation of why this was chosen and the pros versus cons of this method. Was this the method to use?

*Authors’ response: We have added additional discussion of the strengths and weaknesses of reflective practice to the methods and discussion sections.*

**Reviewer comment: **Questions also arise in the explanation of how the focus groups were conducted. For example, more explanation on privacy concerns needs to be discussed. Did you lay ground rules down with the participants? Why was privacy not taken into account prior to conducting the focus groups?

*Authors’ response: Privacy was considered as part of the ethics approval process prior to conducting the focus groups and was not raised as a concern by either the Committee or by participants. From reading your paper, we assume you are most concerned about the potential for others not involved in the study to overhear responses from participants. This was not a major concern in our context, given the discussion topics were not sensitive, but we acknowledge that it may be in other contexts. We have added a point in the discussion to this effect.*

**Reviewer comment: **Also, to help other researchers using online conferencing for focus groups, what are key concerns and positive techniques that should be used for future groups? In terms of results, less data were obtained from the online focus groups, why does this not mean there is a significant difference? Please justify why this is still a sufficient method. Regarding the technical issues, how might these be lessened for others?

*Authors’ response: All of the paragraphs contained in the discussion include our recommendations for future research using web-conferencing, including testing various platforms before deciding on the most appropriate service for a particular study, using headsets to overcome echoing in recording, and aiming to recruit a higher number of participants to allow for the higher withdrawal rates.*

*We do acknowledge that we obtained less data overall in our online groups but highlight that the quality of data was comparable to that obtained face-to-face. We believe that quality is more important than quantity, hence why we argue that there was not a significant difference between the 2 groups. At the very least, it is not an insurmountable issue, as we suggest allowing more time for online groups then you would for face-to-face groups.*

**Reviewer comment: **The major concerns throughout are the aspects that the analysis of this project was not thought of prior to conducting the research. If it was, this needs to be stated more clearly.

*Authors’ response: We acknowledge this as a significant limitation for our study and have added additional information to make the implications of this clearer.*
